# Bioethical issues of preventing hereditary diseases with late onset in the Sakha Republic (Yakutia)

**DOI:** 10.3402/ijch.v73.25062

**Published:** 2014-07-24

**Authors:** Sardana K. Kononova, Oksana G. Sidorova, Sardana A. Fedorova, Fedor A. Platonov, Vera L. Izhevskaya, Elza K. Khusnutdinova

**Affiliations:** 1 Yakutsk Scientific Center of Complex Medical Problems, Siberian Branch of the Russian Academy of Medical Sciences, Yakutsk, Russia; 2 Institute of Natural Sciences, M. K. Ammosov North-Eastern Federal University, Yakutsk, Russia; 3 Research Centre for Medical Genetics of the Russian Academy of Medical Sciences, Moscow, Russia; 4 Institute for Biochemistry and Genetics, Ufa Scientific Centre of the Russian Academy of Sciences, Ufa, Russia

**Keywords:** prenatal diagnosis, bioethics, hereditary diseases, dynamic mutations, genetic counselling, DNA testing

## Abstract

**Background:**

Prenatal diagnosis of congenital and hereditary diseases is a priority for the development of medical technologies in Russia. However, there are not many published research results on bioethical issues of prenatal DNA testing.

**Objective:**

The main goal of the article is to describe some of the bioethical aspects of prenatal DNA diagnosis of hereditary diseases with late onset in genetic counselling practice in the Sakha Republic (Yakutia) – a far north-eastern region of Russia.

**Methods:**

The methods used in the research are genetic counselling, invasive chorionic villus biopsy procedures, molecular diagnosis, social and demographic characteristics of patients.

**Results:**

In 10 years, 48 (76%) pregnant women from families tainted with hereditary spinocerebellar ataxia type 1 and 15 pregnant women from families with myotonic dystrophy have applied for medical and genetic counselling in order to undergo prenatal DNA testing. The average number of applications is 7–8 per year. There are differences in prenatal genetic counselling approaches.

**Conclusion:**

It is necessary to develop differentiated ethical approaches depending on the mode of inheritance, age of manifestation, and clinical polymorphism of hereditary disease.

In the field of gene technologies in medicine, N.A. Holzman and other researchers discuss the following bioethical issues (1,2). There is a large number of publications on this subject. Various DNA tests for patients and carriers of mutant genes will complicate their routine diagnosis because of the genetic heterogeneity of hereditary diseases (3–5). DNA tests for detecting genotypes that increase susceptibility to common diseases (such as familial forms of cancer, coronary heart disease, diabetes) will be available for laboratories of different status. In these situations, evaluation of diagnostic significance of test results will also be extremely difficult (6–8). Screening can cover a large number of people and it will be more difficult to comply with the principle of patient informed consent (PIC). With mass genetic research, the possibility of breaches of confidentiality and discrimination by genetic traits may increase (9–11). There are a lot of ethical issues in the possibility of early presymptomatic diagnosis of individuals–carriers of pathogenic genes, which manifest later in adulthood (12–15). Prenatal diagnosis increases the risk of foetus abuse by making the information about the foetus available in the early stages of development (16–19).

In Yakutia, the term “bioethics” was first introduced in 2000 at the II International Scientific and Practical Conference “Issues of Viluy Encephalomyelitis, Neurodegenerative and Hereditary Diseases of the Nervous System.” In 2000–2002, methods of molecular–genetic diagnosis of hereditary diseases were introduced into public health practice of the Sakha Republic (Yakutia). With the introduction of genomic technologies into public health practice, ethical issues associated with the DNA diagnosis of monogenic diseases arose immediately, which required some specific actions. Particular attention had to be paid to ethical problems of DNA testing for neurodegenerative and neuromuscular hereditary diseases with late onset: spinocerebellar ataxia type 1 (SCA1) and myotonic dystrophy (MD).

Spinocerebellar ataxia type 1 refers to a group of neurodegenerative diseases with late onset. Inheritance is characterized by a high degree of penetrance and the phenomenon of anticipation. Mutation of the *SCA1* gene located on the short arm of chromosome 6 manifests in an uncontrolled increase in the number of *CAG* trinucleotide repeats in the coding region (20). Clinical manifestations of the disease are very diverse; the main ones are slowly progressing incoordination, scanning and explosive speech, presence of cerebellar–pyramidal syndrome, various degrees of degeneration of cerebellum and of its afferent and efferent connections (21).

Rossolimo-Steinert-Batten-Kurshmann myotonic dystrophy (MD) is a hereditary neuromuscular disease characterized by multisystem involvement with a wide variability of clinical symptoms; the main ones of which are myotonia, myopathy, cataracts, cardiomyopathy, endocrine disorders and neuropsychiatric abnormalities in severe cases. The *MD* gene is located on chromosome 19 in the region q13.2–13.3. Mutation of the *MD* gene as well as mutation of the SCA1 gene is of dynamic type and manifests in the expansion of *CTG* repeats (22). There is a high accumulation of the *MD* gene among the Yakut population: 21.3 cases per 100 thousand people (23,24).

Due to the fact that there is still no effective treatment for inherited diseases, prenatal diagnosis remains the only method of prevention. According to international norms and recommendations, prenatal molecular–genetic diagnosis should be carried out with the consent of the family at the earliest possible stages of foetal development (9.5–11 week of pregnancy). At the same time, prenatal DNA testing for diseases with late onset such as SCA1 is considered to be the same as testing a child, which, in accordance with WHO recommendations, should not be carried out until the child reaches adulthood (25).

Phenogenetype features of monogenic diseases, especially their late onset, require a balanced and cautious approach to prevention, with bioethical principles as fundamental ones.

The article presents data on prenatal DNA testing for hereditary diseases with late onset and approaches to dealing with bioethics in the field of genetic technology in healthcare of the Sakha Republic (Yakutia).

## Methods

### Setting

Yakutia or the Sakha Republic is one of the largest northern regions of the Russian Federation with a territory of 3,083,523 km^2^ and a population of about 955,000 people. Geographical remoteness from major Russian cities, poor infrastructure and harsh climatic conditions inhibit human settlement in the area, resulting in population density of 0.31 pers./km^2^. The indigenous population, represented by the Yakuts, Russians, Evens and Evenks, lives in small cities and towns. There are 34 administrative districts (uluses) in the Sakha Republic (Yakutia). According to the National Census of 2010, the Yakut population is 466,000 people. The capital of Yakutia is the City of Yakutsk with about 280,000 people, which is almost a third of the entire population (26).

### Patients

We have used the data of the Republican genetics registry of hereditary and congenital abnormalities of the Sakha Republic (Yakutia). The registry has a record of 105 hereditary diseases (24). Compared to global data, the prevalence of neurodegenerative and neuromuscular monogenic diseases with late onset is very high among the numerically small Yakut population. For example, the frequency of SCA1 is 36.8 per 100,000 population (27); myotonic dystrophy – 21.3:100,000 (23).

In 10 years of observation, 63 pregnant women from families tainted with hereditary dynamic mutations have applied for prenatal medical genetics counselling; 48 (76%) of them were tainted with SCA1, and 15 with MD (Table I). The age of these women ranged from 18 to 40; all women were Sakha by origin. The average age was 26 years. Patients with SCA1 and MD mainly came from the regions of dynamic accumulation of mutations in Yakutia: Ust-Aldan, Tatta, Megino-Kangalassky, Amginsky, Suntarsky and Nyurbinsky uluses. Thirteen women with SCA1 and 5 with MD lived in Yakutsk. Social and demographic characteristics of the patients were studied using medical records.

**Table I:** Some characteristics of the patients from families affected with a spinocerebellar ataxia type 1 and myotonic dystrophy

Patients		

Spinocerebellar ataxia type 1	Total (n=48)	%
Middle age	26.2	
Education (higher/secondary)	29/11	60/23
Hereditary transfer in the area: father/mother/husband	15/15/13	50/50/27
Family decision (consent/refusal)	26/22	55/46
Myotonic dystrophy	Total (n=15)	%

Middle age	25.2	
Education (higher/secondary)	3/12	20/80
Hereditary transfer in the area: father/mother/husband	5/8/2	19/62/13
Family decision (consent/refusal)	13/2	85/15

### Instrumentation

Genetic counselling of pregnant women with SCA1 and MD, referred to PD, was done in several stages:

- Medical history, genealogy detailing on the registry of hereditary diseases, ultrasound control screening, informing about the disease, the possibilities of DNA testing and prenatal diagnosis (risks vs. benefits), obtaining PIC to conduct DNA testing for mutation carriers (for those who have not undergone such testing earlier) and PD procedures;
- Chorionic villus biopsy procedure and monitoring of pregnant women at daytime patient care department;
- Counselling on the results of PD. In case of a positive result of DNA testing and the woman's decision to have an abortion, receiving PIC for this procedure.

Trans-abdominal invasive procedure of chorionic villus biopsy/platsentocentesis was performed on patients at different stages of pregnancy (from a minimum period of 9 weeks up to a maximum of 22 weeks). DNA was extracted from chorionic villae using standard methods and further detection of mutation in a foetus was done using polymerase chain reaction (PCR) as described by Mathew (28).

Amplification of the *SCA1* gene *CAG*-repeats for SCA1 diagnosis was performed with PCR using primers Rep-1 and Rep-2, as suggested by Orr et al. (29). PCR was carried out on the Gene Amp PCR System 9600 device (Perkin Elmer). Amplification products were separated in a 2% agarose gel with bromide ethidium and then visualized under UV light. This method allows to clearly identify the mutant elongated allele as a DNA fragment with the number of *CAG*-repeats of more than 40.

When DNA testing for MD, primers described by Brook were used for PCR (30). Amplification products were separated in an 8% polyacrylamide gel. The size of the alleles was determined using DNA test samples with a known number of repeats.

## Results

In the period between 2002 and 2013, 48 pregnant women from families tainted with SCA1 applied for prenatal diagnosis. Thirty women were carriers of the mutation, of which 15 (50%) had inherited it from their mothers, and 15 (50%) from their fathers. The vast majority (96%) of mutation carriers had no clinically significant symptoms of the disease, that is, they were subjected to the pre-symptomatic DNA diagnosis. Five pregnant women had healthy but had partners (husbands) from tainted families (Table I). Twenty-six (55%) women gave their consent for prenatal DNA testing of the foetus. According to the results of DNA diagnostics, 14 (54%) cases showed the absence of mutations in the foetus, all of these pregnancies were continued and ended in childbirth. In 12 foetuses (47%), the presence of abnormally elongated allele was discovered. Ten women expressed a desire to terminate the pregnancy and 2 refused to undergo an abortion after a positive DNA test.

The most important aspects in the prenatal diagnosis of hereditary diseases are the term of pregnancy at the moment of applying for genetic counselling and pregnancy termination in case of abnormal foetus detection. The above is especially important in case of diseases with late onset. In our practice, 26 (56%) patients applied for counselling in the first trimester of pregnancy and 20 (43%) women were in the later stages. Termination of a pregnancy of up to 12–14 weeks was performed in 5 women, and up to 22 weeks in 5 patients. In addition, there were 2 cases when women with foetuses – mutation carriers – made a decision not to terminate pregnancy. In our opinion, it is necessary to raise the question of prohibiting the termination of a pregnancy with *SCA1* mutation in the second and third trimester, as it not only entails serious moral and psychological consequences for women but also has obstetric complications with adverse consequences for the patients’ future reproductive function.

In the period between 2002 and 2010, 15 families tainted with MD asked for prenatal diagnostics. Unlike patients with SCA1, most women with MD – 8 (62%) – had clinical signs of MD at the moment of application for PD. Of the total, 5 (19%) had inherited the disease from their mothers and 8 (62%) from their fathers, and 2 clinically healthy women had partners from families tainted with MD (Table I).

## Discussion

In order to prevent hereditary diseases, different states adopt programs of presymptomatic testing and prenatal diagnosis. For example, in 1999–2009, in Brazil, in accordance with such a program, 184 individuals were tested, of which 80% had a risk of spinocerebellar ataxia (SCA) – SCA3 (31). A similar situation to the one in the Sakha Republic (Yakutia) with high prevalence of hereditary neurodegenerative diseases exists in Cuba in relation to spinocerebellar ataxia type 2 (SCA2) (32). Presymptomatic diagnosis was carried out in analysed population (n=768) and 223 carriers of SCA2 were identified (33). In the Sakha Republic (Yakutia) in 2000–2013, 1,841 people were tested for SCA1 and mutation was identified in 606 individuals, of which 100 (20%) are asymptomatic carriers of SCA1 (34).

Prenatal genetic counselling poses a number of ethical dilemmas for doctors and families, first of all on the issue of making a decision (35). In comparison with people with SCA1, the percentage of people with MD who refused PD was less – 2 (15%). This is attributed to the fact that the woman's consent to undergo prenatal diagnosis is heavily influenced by relatives. It is known that among the variety of clinical signs, patients with MD manifest general asthenia and lowering of intelligence to some extent, which makes it hard for a pregnant woman with MD to make an independent decision on the need of PD. Similar cases are noted in existing publications in connection with prenatal genetic counselling of teenage girls. The authors identified differences in methods of communicating with teenagers and adult women. Teenagers find it hard to understand information regarding risks to foetus. In the Sakha Republic (Yakutia), complicated ethical and legal issues relating to prenatal diagnosis for MD have arisen as well as conditions for violating the rights of the patients (36). Many patients with MD who have an officially recognized disability are legally competent, that is, they have no legal guardians, and as such have the right to make independent decisions concerning PD and to sign informed consent forms. However, in most cases, patients with MD are highly dependent on their relatives caring for them and their children. It is quite reasonable that in such a situation relatives actively influence the decisions made by MD patients. The principle of confidentiality within a family also loses its significance. It is interesting that most women who have applied for PD were representatives of two large families. Here, a significant role was played by the most active members of families, usually women, who informed the whole family about the possibilities of prenatal diagnosis.

Prenatal diagnosis of diseases with dynamic mutations was first carried out in 2002 at the prenatal diagnosis department of genetic counselling. Introduction of DNA testing for hereditary diseases into the practice of medical and genetic counselling in Yakutia gave reason to believe that a demand for prenatal diagnostic procedure would be created among the patients from tainted families regardless of nosology. Especially high hopes were connected with the use of SCA1 prenatal diagnosis as a basic tool for preventing births of new *SCA1* mutation carriers. Thus, in the early years of employing this new technique in the practice of regional medical genetic counselling, clinical geneticists expressed the opinion about the appropriateness of compulsory DNA testing in order to proactively identify SCA1 carriers, bearing in mind the high social importance of the disease in the Sakha Republic (Yakutia). However, after numerous discussions, it was decided that we should strictly adhere to international ethical principles (non-directive counselling, respect for any decision of a patient, voluntary testing, confidentiality) related to prenatal testing for diseases with late onset. With this approach we should expect an average of 7–8 applications per year for prenatal genetic counselling from families with SCA1. It is possible that the long history of existence of *SCA1* mutation among the Yakut population led to families with ataxia developing a certain attitude towards the disease. Therefore, the small number of applications cannot be explained by the low level of awareness about the possibilities of PD or financial difficulties (PD procedure and DNA testing are free of charge). For example, of the 63 pregnant women who have applied for medical genetic counselling in 10 years, about a half refused to undergo PD.

We have found some differences in the approaches to genetic counselling among the families tainted with diseases with dynamic mutations SCA1 and MD. There is a difference in the level of education of women who have applied for PD. Usually women with *SCA1* mutations have higher or secondary special education and are socially active and adapted, while women with MD mostly have secondary or school education. At the time of applying for medical genetic counselling, in most cases, patients with MD were already sick. Therefore, they were socially less adapted, more passive and immature, and were often accompanied by their healthy family members. In making morally difficult reproductive decisions (acceptance/refusal of PD, consent/refusal of abortion), patients with SCA1 made most decisions independently, the number of motivated refusals to undergo PD was higher, just like the number of consents to an abortion in case of positive DNA test results. In contrast to the above, women with MD refused to undergo PD less, but more often refused to terminate a pregnancy in case of positive DNA test results (often these refusals were not motivated). Since doctors could not affect the situation in such cases, in 6 years 2 children were born with congenital form of MD.

## Conclusions

The use of genetic technologies in the medical practice of the Sakha Republic (Yakutia) raises a number of bioethical issues relating to prenatal DNA testing for hereditary diseases with late onset. When employing prenatal diagnosis for diseases with late onset it is necessary to apply an ethical right to refuse to terminate pregnancy in the second and third trimesters in order to prevent complications and save the pregnant woman's reproductive function. The characteristic feature of MD genetic counselling, unlike SCA1, is the inevitability of involving relatives in the process of informing the pregnant woman and making difficult decisions for the family. The principle of confidentiality within the family loses its significance. This fact should be taken as a valid ethical norm for patients with MD applying for medical genetics help.
